# Renal artery sympathetic denervation: observations from the UK experience

**DOI:** 10.1007/s00392-015-0959-4

**Published:** 2016-01-22

**Authors:** Andrew S. P. Sharp, Justin E. Davies, Melvin D. Lobo, Clare L. Bent, Patrick B. Mark, Amy E. Burchell, Simon D. Thackray, Una Martin, William S. McKane, Robert T. Gerber, James R. Wilkinson, Tarek F. Antonios, Timothy W. Doulton, Tiffany Patterson, Piers C. Clifford, Alistair Lindsay, Graeme J. Houston, Jonathan Freedman, Neelan Das, Anna M. Belli, Mohamad Faris, Trevor J. Cleveland, Angus K. Nightingale, Awais Hameed, Kalaivani Mahadevan, Judith A. Finegold, Adam N. Mather, Terry Levy, Richard D’Souza, Peter Riley, Jonathan G. Moss, Carlo Di Mario, Simon R. Redwood, Andreas Baumbach, Mark J. Caulfield, Indranil Dasgupta

**Affiliations:** Department of Cardiology, Royal Devon and Exeter NHS Foundation Trust, University of Exeter, Barrack Road, Exeter, EX2 5DW UK; International Centre for Circulatory Health, National Heart and Lung Institute, Imperial College London, London, UK; Barts BP Centre of Excellence, Barts Heart Centre, St Bartholomew’s Hospital, W Smithfield, London, EC1A 7BE UK; William Harvey Research Institute, Barts NIHR Cardiovascular Biomedical Research Unit, Queen Mary University of London, London, E1 4NS UK; Royal Bournemouth Hospital, Castle Lane East, Bournemouth, BH7 7DW UK; Institute of Cardiovascular and Medical Sciences, University of Glasgow, Glasgow, G12 8TA UK; University of Bristol, Bristol, UK; Hull and East Yorkshire Hospitals NHS Trust, Hull York Medical School, Castle Road, Hull, HU16 5JQ UK; Department of Clinical Pharmacology, School of Clinical and Experimental Medicine, University of Birmingham, Birmingham, UK; Sheffield Kidney Institute, Northern General Hospital, Herries Road, Sheffield, S5 7AU UK; East Sussex Healthcare NHS Trust, Eastbourne, East Sussex UK; University Hospital Southampton NHS Foundation Trust, Tremona Road, Southampton, SO16 6YD UK; St George’s University Hospitals NHS Foundation Trust, London, SW17 0QT UK; East Kent Hospitals NHS Foundation Trust, Kent and Canterbury Hospital, Ethelbert Road, Canterbury, CT1 3NG UK; Cardiovascular Division, Rayne Institute, BHF Centre of Research Excellence, Kings College London, St Thomas’ Hospital, Westminster Bridge Road, London, SE1 7EH UK; Buckinghamshire Healthcare NHS Trust, Queen Alexandra Road, High Wycombe, Bucks HP11 2TT UK; NIHR Cardiovascular BRU Royal Brompton & Harefield NHS Foundation Trust, London, SW3 6NP UK; Division of Cardiovascular and Diabetes Medicine, Medical Research Institute, University of Dundee, Dundee, UK; Department of Interventional Radiology, Heart of England NHS Foundation Trust, Birmingham, UK; Sheffield Vascular Institute, Northern General Hospital, Herries Road, Sheffield, S5 7AU UK; University of Birmingham, Birmingham, UK; Bristol Heart Institute, University Hospitals Bristol NHS Foundation Trust, Bristol, BS2 8HW UK; Department of Renal Medicine, Royal Devon and Exeter NHS Foundation Trust, Barrack Road, Exeter, EX2 5DW UK; Radiology Department, Queen Elizabeth Hospital Birmingham, University Hospital Birmingham NHS Trust, Edgbaston, Birmingham, B15 2TH UK; Queen Elizabeth University Hospital, Glasgow, G11 6NT UK; Renal Unit, Birmingham Heartlands Hospital, School of Clinical and Experimental Medicine, University of Birmingham, Birmingham, UK

**Keywords:** Hypertension, Sympathetic nervous system, Catheter ablation, Aldosterone

## Abstract

**Background:**

Renal denervation (RDN) may lower blood pressure (BP); however, it is unclear whether medication changes may be confounding results. Furthermore, limited data exist on pattern of ambulatory blood pressure (ABP) response—particularly in those prescribed aldosterone antagonists at the time of RDN.

**Methods:**

We examined all patients treated with RDN for treatment-resistant hypertension in 18 UK centres.

**Results:**

Results from 253 patients treated with five technologies are shown. Pre-procedural mean office BP (OBP) was 185/102 mmHg (SD 26/19; *n* = 253) and mean daytime ABP was 170/98 mmHg (SD 22/16; *n* = 186). Median number of antihypertensive drugs was 5.0: 96 % ACEi/ARB; 86 % thiazide/loop diuretic and 55 % aldosterone antagonist. OBP, available in 90 % at 11 months follow-up, was 163/93 mmHg (reduction of 22/9 mmHg). ABP, available in 70 % at 8.5 months follow-up, was 158/91 mmHg (fall of 12/7 mmHg). Mean drug changes post RDN were: 0.36 drugs added, 0.91 withdrawn. Dose changes appeared neutral. Quartile analysis by starting ABP showed mean reductions in systolic ABP after RDN of: 0.4; 6.5; 14.5 and 22.1 mmHg, respectively (*p* < 0.001 for trend). Use of aldosterone antagonist did not predict response (*p* > 0.2).

**Conclusion:**

In 253 patients treated with RDN, office BP fell by 22/9 mmHg. Ambulatory BP fell by 12/7 mmHg, though little response was seen in the lowermost quartile of starting blood pressure. Fall in BP was not explained by medication changes and aldosterone antagonist use did not affect response.

**Electronic supplementary material:**

The online version of this article (doi:10.1007/s00392-015-0959-4) contains supplementary material, which is available to authorized users.

## Introduction

Hypertension contributes to 62 % of all strokes, 49 % of global heart disease burden and causes an estimated 7.1 million deaths a year [1, 2]. In most real-world datasets, however, fewer than 50 % of subjects are at target despite a range of pharmacological options. The reasons for this are complex, but it seems clear that new strategies for the management of uncontrolled hypertension are required [3, 4].

Reduction of sympathetic outflow is one proposed alternative to drug treatment for reducing high blood pressure. Invasive surgical sympathectomy was shown to lead to significant blood pressure reductions over 70 years ago; however, this procedure was abandoned due a high complication rate [5]. Renal artery sympathetic denervation aims to more selectively abrogate efferent and afferent sympathetic nerve signals to and from the kidney, to reduce sympathetic nervous activity and therefore blood pressure [6, 7].

Early observational data and open-label, randomized studies suggested substantial reductions in blood pressure following a single percutaneous procedure [8, 9]. Reduction in sympathetic tone through RDN also appeared to be associated with potential beneficial effects on hypertension end-organ effects and in other conditions where sympathetic drive may modulate the disease condition [10–16].

More recently, a rigorously conducted randomized trial added renal denervation (RDN) to a stepped anti-hypertensive drug program and demonstrated incremental blood pressure lowering with RDN [17]. However, a sham-controlled trial of RDN (symplicity HTN-3) failed to meet its primary efficacy endpoint [18]. The procedure met its safety endpoint, but similar reductions in blood pressure were seen between the renal denervation group and the sham-control group. Secondary sub-analyses of the trial dataset by the authors have suggested potential confounders, principal amongst them being that fewer than 6 % of patients received per-protocol bilateral retrograde spiral ablation [19].

The efficacy of this technology therefore remains uncertain and further randomized trials are required. In the meantime, more data are required on the nature of the patients who have already undergone the procedure, their response to treatment and identification of factors that may affect subsequent blood pressure response. Such data would better inform the design, conduct and interpretation of future trials.

This article reports the UK experience with RDN for treatment-resistant hypertension. It examines the nature of the blood pressure response seen on ambulatory monitoring and the impact of drug changes post denervation on the results. Finally, this study examines the interaction of RDN with the use of aldosterone antagonists.

## Methods

At the time of instigation of the UK Renal Denervation Affiliation (May 2014), background research by the study team identified 21 centers that had performed five or more procedures in the UK. The aim of this study was to give as complete as possible a representation of the UK national experience with RDN to date, by collating retrospective data from all procedures performed on patients with uncontrolled hypertension.

Each center was contacted by email and/or telephone and invited to participate in the registry. Eighteen of those centers agreed to contribute and provided the data that form the basis for this manuscript. These data represent the results of cases performed for treatment-resistant hypertension, as defined by prior international consensus statements [7] and in accordance with the Joint UK Societies consensus statement [20]. A small number of cases were excluded as they were performed for other indications as part of ongoing clinical trials (e.g. heart failure; sleep apnea; acknowledged non-compliance with medications).

Anonymized data were collated locally using a spreadsheet specifically designed for the study and then submitted to a central coordinating center (Exeter, UK), where it was analyzed. The project was independent of any financial support from industry and is an exclusively investigator-led initiative.

‘Responders’ to RDN are defined according to prior convention [21], by a reduction in office systolic blood pressure of ≥10 mmHg and reduction in daytime ambulatory systolic blood pressure fall of ≥5 mmHg from baseline to follow-up [18]. Absence of normal nocturnal dipping profile on pre-procedural ABP was defined as a fall in nighttime systolic ABP of <10 %. The lattermost BP readings available are reported.

Data are presented as mean ± standard deviation (SD) unless stated. Between group variations were analyzed using Chi Square test for categorical variables and where normality was demonstrated, a *T* test or ANOVA were used for continuous variables. Logistic regression models were used to examine the interaction of aldosterone antagonist use with blood pressure response following RDN after adjustment for factors previously thought to interact with response to RDN from prior literature. These models were also used to look for other potential baseline predictors of blood pressure changes after RDN. A *p* value of <0.05 was considered statistically significant. Statistical analyses were performed using SPSS software v20.0 (SPSS Inc. Chicago, IL).

## Results

Results from 253 subjects, treated in 18 centers using five different technologies, are included in this analysis. These include Symplicity Flex [*n* = 204 (81 %)]; Symplicity Spyral [*n* = 10 (4 %)]; Boston Vessix [*n* = 3 (1 %)]; St Jude Enlightn [*n* = 26 (10 %)] and Covidien Oneshot [*n* = 10 (4 %)]. Mean age of patients was 57 years; 53 % were female; 88 % Caucasian and 26.5 % were diabetic (Table 1).

**Table 1:** Demographic data

Demographic data	Mean	SD
Age	57	11.8
Serum creatinine	93	36.3
eGFR (MDRD method)	69	21.4
BMI	32	6.4

Demographic data	*n*	%
Female	133	53
Caucasian ethnicity	223	88.1
Diabetes	67	26.5
Previous CVA/TIA	61	24.1
Previous myocardial infarction	38	15.0
Symptomatic IHD—previous MI/chronic stable angina	58	22.9
Heart failure	12	4.7
Proteinuria	60	23.7

*SD* standard deviation

Eighty-six percent of patients were seen in a dedicated hypertension clinic with each patient being reviewed by an average of 1.6 hypertension specialists. These included cardiologists, nephrologists, clinical pharmacologists and endocrinologists. All patients had anatomical screening of their renal arteries prior to their RDN procedure.

The majority of patients had pre-procedural ambulatory blood pressure monitoring performed (73.5 %) and detailed assessments to rule out secondary hypertension before the procedure (screening details are shown in Table 2).

**Table 2:** Screening process

Screened by		
Mean number of hypertension specialists seen	1.6	SD (0.7)
Nephrologist	115	45 %
Cardiologist	168	66 %
Clinical pharmacologist	91	36 %
Endocrinologist	38	15 %
Screening process		
Diet and Lifestyle re-reviewed	250	99 %
Hypertension managed within dedicated hypertension clinic	217	86 %
Pre-procedural renal CTA/MRA	220	87 %
Pre-procedural renal CTA/MRA/USS	253	100 %
Documented screening results for Cushing’s disease	113	45 %
Documented screening results for phaeochromocytoma	202	80 %
Documented screening results for Conn’s syndrome	159	63 %

*SD* standard deviation

Mean office BP before procedure was 185/102 mmHg (SD 26/19; *n* = 253) with an average daytime ambulatory blood pressure (ABP) of 170/98 mmHg (SD 22/16; *n* = 186). Fifty-eight percent of the cohort had loss of normal nocturnal dipping on ABP. The median number of antihypertensive drugs prescribed before RDN was 5.0 including 96 % ACEi/ARB; 86 % thiazide or a loop diuretic and 55 % aldosterone antagonist prescription at the time of denervation (Tables 3, 4).

**Table 3:** Pre-procedural blood pressure

Blood pressure pre-procedure	*n* = 253	SD
Office Systolic BP (mmHg)	185	26
Office Diastolic BP (mmHg)	102	19

Blood pressure pre-procedure	*n* = 186	SD
Daytime systolic ABP (mmHg)	170	22
Daytime diastolic ABP (mmHg)	98	16
Night-time systolic ABP (mmHg)	154	26
Night-time diastolic ABP (mmHg)	86	18
Loss of normal nocturnal dipping profile (%)	58	

*SD* standard deviation

**Table 4:** Medications taken by the cohort at the time of denervation

Medications at time of denervation	
Median number of medications per patient	5
Renin-angiotensin system blocker^a^ (%)	96
B-blocker (%)	65
Calcium channel blocker (%)	73
Diuretic (any) (%)	95
Diuretic-aldosterone antagonist (%)	55
Diuretic-thiazide (%)	52
Diuretic-loop (%)	34
Diuretic-amiloride (%)	2
Alpha-blocker (%)	50
Moxonidine (%)	17
Minoxidil (%)	7
Hydralazine (%)	6
Methyldopa (%)	6
Oral nitrate/nicorandil (%)	5
Clonidine (%)	3

^a^ACE Inhibitor or angiotensin receptor blocker or direct renin inhibitor

Clinical follow-up was available in 90 % of subjects, with mean duration of office BP follow-up of 11 months. Mean post-procedural office BP was 163/93 mmHg, representing a fall in office BP following RDN of 22/9 mmHg.

ABP data were available in 70 % of cases post-procedure, at a mean follow-up duration of 8.5 months. Average daytime ABP at the end of follow-up was 158/91 mmHg, representing a fall in daytime ABP following RDN of 12/7 mmHg. Average number of antihypertensive agents added per patient was 0.36. Average number of agents withdrawn per patient was 0.91 (Tables 5, 6). Drug dose changes appeared to be balanced across the cohort, in terms of dose escalation and reductions.

**Table 5:** Follow-up blood pressure data

	Mean	SD
Follow-up office BP data (*n* = 228; 90 %)		
Duration of follow-up	11.0	6.7
Systolic BP	163	28
Diastolic BP	93	19
Mean fall in cohort office SBP (mmHg)	22	29
Mean fall in cohort office DBP (mmHg)	9	19
Follow-up ABP data (*n* = 177; 70 %)		
Duration of follow-up	8.5	4.0
Daytime systolic BP	158	25
Daytime diastolic BP	91	17
Night-time systolic BP	145	26
Night-time diastolic BP	83	17
Mean fall in cohort daytime systolic ABP (mmHg)	12	
Mean fall in cohort daytime diastolic ABP (mmHg)	7	

*SD* standard deviation

**Table 6:** Drug changes following RDN procedure

	*n*	%
Drugs added since procedure		
0	165	65
1	42	17
2	15	6
3	2	1
4	1	0
Data not available	28	11
Drugs stopped since procedure		
0	127	50
1	45	18
2	26	10
3	10	4
4	11	4
5	3	1
6	2	1
7	1	0
Data not available	28	11
Average number of anti-hypertensive drugs added since procedure (per patient)	0.36	
Average number of anti-hypertensive drugs stopped since procedure (per patient)	0.91	
Drug dose changes		
Average number of drug doses up-titrated per patient	0.21	
Average number of drug doses decreased per patient	0.17	
Patients with no changes in drug numbers or drug doses	80	
Patients with changes in either drug numbers or drug doses	128	
Drug dose changes not available	45	

**Fig. 1:**
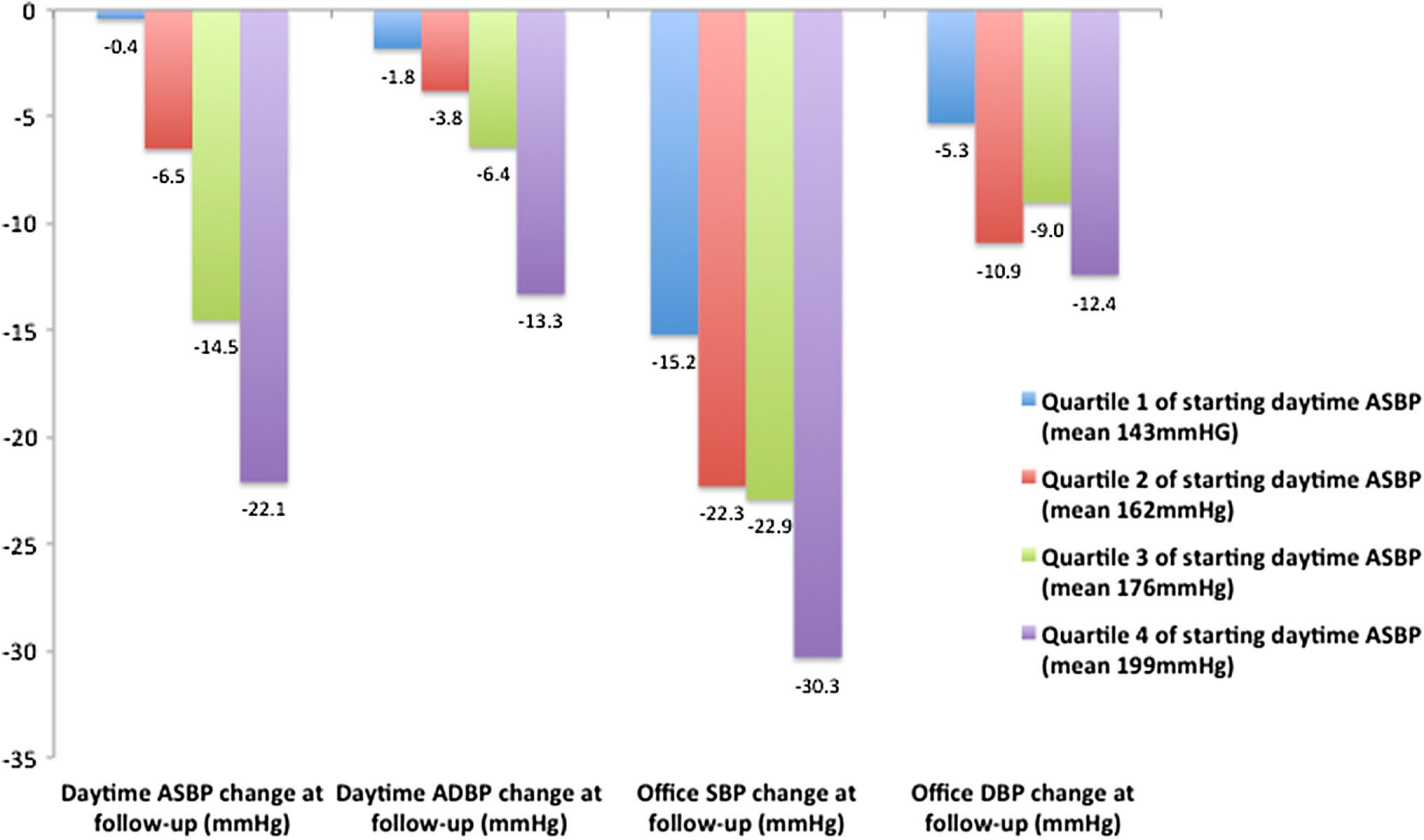
Change in daytime ambulatory systolic blood pressure (ASBP), daytime ambulatory diastolic blood pressure (ADBP), office systolic BP (SBP) and office diastolic BP (DBP) to RDN by quartile analysis of starting daytime ambulatory SBP

Figure 1 shows BP response to RDN according to quartile of baseline daytime ambulatory systolic BP. Baseline mean daytime ambulatory systolic BP (ASBP) from quartile 1 to 4 was: 142, 162, 176 and 199 mmHg, respectively. Number of antihypertensive drugs per quartile did not significantly differ (*p* > 0.2). At 8.5 months follow-up, the mean reductions in daytime ASBP by quartile of starting daytime ASBP were: 0.4; 6.5; 14.5 and 22.1 mmHg, respectively (*p* value for quartile trend <0.001).

Office systolic BP (OSBP) also exhibited significant falls across each of the four quartiles (*p* = 0.001 for quartile trend), but in the lowest quartile, this was not matched by a statistically significant ASBP response. Overall, 65 % patients were defined as responders, with a ≥10 mmHg fall in OSBP. A similar percentage (62 %) had a ≥5 mmHg fall in daytime ASBP.

Use of aldosterone antagonist at the time of RDN did not predict the degree of blood pressure response (*p* > 0.2 as univariate predictor). This remained the case after adjustment for the following potential confounders: age, gender, diabetes, estimated glomerular filtration rate (eGFR), number of drugs taken and starting office blood pressure (*p* > 0.2). There remained no association when ASBP was substituted for OSBP within the model. The only baseline characteristic that predicted subsequent fall in BP after RDN was blood pressure, as measured by office or ABP.

## Discussion

This investigator-led study reports the results of RDN procedures for 253 people with treatment-resistant hypertension, performed in 18 UK specialist centers using five different technologies. It shows significant reductions in both office and daytime ambulatory blood pressure of 22/9 and 12/7 mmHg, respectively, after a mean follow-up period of 11 months (*p* < 0.001 for both findings). This blood pressure reduction does not appear to be related to changes in anti-hypertensive medications made after denervation and use of aldosterone antagonists did not affect blood pressure response following the procedure. Patients in the two highest quartiles of daytime ambulatory systolic blood pressure at baseline exhibited significant ambulatory blood pressure reductions, whilst those in the lowest quartile exhibited little response.

To date, in excess of 10,000 patients worldwide have been treated with renal artery denervation [22]. Observational studies and open-label randomized controlled studies have suggested significant falls in blood pressure following treatment, with a particularly well-designed study (DENER-HTN) suggesting incremental benefit from RDN when applied over and above stepped anti-hypertensive therapy [9, 17, 23]. However, a randomized, sham-controlled trial (Symplicity HTN-3) failed to meet its primary efficacy endpoint, with blood pressure reductions in the denervated group matched by blood pressure falls in the sham-control arm [18].

There has been extensive commentary to date on the possible reasons why HTN-3 failed [22, 24, 25]. The first and most obvious reason is that the technology may be ineffective. However, the HTN-3 investigators have recently released data suggesting that the technique used in HTN-3 may have been sub-optimal, with only 6 % of subjects treated with the recommended bilateral retrograde spiral technique [19]. Clearly, sub-optimal denervation could have confounded the trial [26].

Other commentators have suggested that the failure of HTN-3 may have been catheter specific [27]. Further suggestion has been made of the role of ethnicity, the screening process, drug changes and lack of formal testing for drug adherence within that trial. These theories represent post hoc speculation in response to an unexpected negative result and therefore have to be considered with caution.

Sound physiological principles and surgical precedent underpin the field of RDN, which is operating in an area of medicine with substantial unmet need; the technology remains promising, appears safe [18, 28–30] and further randomized trials are required.

In this report, the UK Renal Denervation Affiliation details the results from 18 centers, each of which had performed more than five cases to date. Case selection was typically in accordance with the Joint UK Societies Consensus statement on RDN, which recommended strict criteria for patient selection [24]. These included: OSBP >160; systolic ASBP >150 on ≥3 antihypertensive agents (or in step 4 of the UK guideline [31]); exclusion of white-coat or secondary hypertension and patient selection by multidisciplinary teams of hypertension specialists and interventionists.

These data present a near-comprehensive national experience of the use of RDN technology, with over 85 % of experienced UK centers represented. The mean number of cases performed per center within this registry was 15 (SD 6.7). By comparison, the mean number of cases per center in HTN-3 was 4.1 [18].

Results show a fall in office BP of 22/9 mmHg at 11 months. Clearly, such data are open label and therefore have to be observed with caution, but this is a well screened and treated cohort, having previously been looked after by an average of 1.6 specialists with a named interest in hypertension. More than 85 % of subjects were managed in dedicated hypertension clinics prior to their denervation and relatively few drug increases were attempted after their procedure, despite the severity of their hypertension, suggesting a stable drug regimen was in place prior to their treatment.

Three times as many drugs were withdrawn following denervation as were added, with otherwise balanced dose-titrations observed across the cohort. This suggests that drug changes following RDN may have served to reduce the observed treatment effect of denervation, rather than magnify it. Clearly, however, whatever drug regimens were pursued, office blood pressure is prone to unconscious confounding, as described by others [32, 33]. Alternate methods of assessment of the success (or otherwise) of the technology are required.

Ambulatory blood pressure is a stronger predictor of outcome than office or home blood pressure readings and a better measure of true blood pressure than office readings. It is less prone to variability or confounding and makes pseudoresistant hypertension unlikely [34].

In this clinical cohort, a high proportion of patients were assessed using ABP. Some patients could not tolerate the repeated measures of ABP, which is understandable, given that some had very high blood pressures (office SBP >240) at the time of assessment. Despite this, follow-up ABP results were available in over 70 % of subjects.

Importantly, our cohort showed good pre-procedural approximation between office and ambulatory BP (pre-procedural office BP 186/102 mmHg; pre-procedural ABP of 170/98 mmHg) suggesting little white-coat element across the cohort.

Furthermore, subjects were prescribed an average of five medications at the time of their procedure, with a relatively high use (55 %) of aldosterone antagonists. To our knowledge, this is the highest proportion of subjects using aldosterone antagonists in any population of RDN patients reported to date. Despite these proactive drug strategies, mean pre-procedural daytime ABP remained 170/98 mmHg, which is, to our knowledge, as severe a cohort of hypertensives as have been studied with denervation to date.

Prior studies suggest an important role for aldosterone antagonism in the area of treatment-resistant hypertension [35]. The recently published PATHWAY-2 trial has confirmed spironolactone’s superior efficacy as a ‘step-four’ drug in hypertension pathways, when compared to B-Blockers and doxazosin. Furthermore, spironolactone has been proposed as an alternative to denervation, or a possible synergistic agent in blood pressure reduction following RDN [19, 36–38].

In this UK cohort of subjects, the use of aldosterone antagonists was not associated with a difference in blood pressure after RDN but this does not in any way question the efficacy of spironolactone in a resistant population. In this population, RDN was reserved for patients who were resistant to all recommended treatments and therefore we are observing a ‘treat to target’ effect, whereby other drugs are being used to compensate for the presence or absence of a spironolactone effect before a decision on RDN is made. Aldosterone antagonism use was not randomized within our cohort and therefore, by definition, the groups were not balanced and cannot be directly compared for efficacy of the drug. Rather, we report this to establish whether spironolactone acts as a predictor of blood pressure fall after RDN and it does not.

The pattern of ABP quartile response to RDN is an interesting finding from this study. The relationship between starting office blood pressure and magnitude of fall in BP has been previously described for office BP and we replicate this finding with our data. It has been suggested that this could represent a regression to the mean artefact, but ABPM is more resistant to that bias than office BP [39]. The large GLOBAL registry has also suggested an association of starting ABP with magnitude of fall in BP, but quartiles of response were not reported [29].

Given the strength of the association of fall in ASBP with ‘true’ starting BP observed in our cohort (as defined by daytime ASBP) these findings suggest that the most likely way to demonstrate the effectiveness of RDN in a randomized, blinded trial, where unconscious confounding of office response cannot bias the result and treatment effect sizes tend to be smaller [33], may be to test it in a cohort of patients with significant hypertension on ABP, rather than OBP.

Upcoming trials in the field of renal denervation have been proposed in patients with moderate hypertension (ABP of 140-170). Such patients most closely approximate to subjects in quartiles 1 and 2 of our study. Given that these trials are sham-controlled, we may expect to see results closer to those of daytime ABP, rather than open-label office pressure [32]. Rigorous control of potential confounders will therefore be important to pick up a true treatment effect size if these BP reductions are replicated.

Given the morbidity and mortality associated with uncontrolled hypertension, the need for treatment options over and above medications remains apparent, especially for those with few remaining medical options. Whilst we await further randomized trials in this area, results from this cohort of UK subjects with severe, treatment-resistant hypertension and few remaining medical options suggest that, on average, blood pressure control improved following RDN, especially in those with ambulatory blood pressure readings in the highest range.

### Study limitations

This is an open-label retrospective registry with no outside funding source and therefore no independent verification of results was obtained. It is therefore limited by this study design. However, data quality appeared good, as supported by the relatively high frequency of reporting of ABP results and the close correlation between office BP and ABP results. Results also appear consistent across 18 sites.

This study did not mandate measures of adherence to prescribed medications and therefore variable levels of compliance pre- and post-procedure could have had a confounding impact on results. However, there is no reason to suspect changes in behavior occurred according to starting blood pressure and subjects within each quartile of starting ABP were prescribed similar numbers of pharmacological agents. Recent data have also shown blood pressure reductions after RDN in proven compliant subjects [40].

Future studies of RDN should, though, incorporate a direct assessment of adherence, such as urine antihypertensive drug analysis to look for confounding from variable drug compliance on results. Ultimately, however, novel measures of blood pressure reduction are needed for both adherent and non-adherent subjects and both these groups require study within separate, dedicated, randomized trials.

Reporting of ABP is not universal. ABP was tried in almost all patients within this cohort, but with a starting BP of >200 mmHg in many of these patients, the device was not tolerated for a 24-h period. Furthermore, many of these subjects travelled long distances to an RDN center for review and therefore repetition by the performing center was not feasible. Despite these limitations and the absence of reimbursement for RDN in the UK, 73 % use is a higher rate of use of ABP than in other funded registries to date.

## Conclusion

This real-world study demonstrates that renal artery sympathetic denervation is associated with a significant reduction in both office and ambulatory blood pressure in well-characterized subjects with treatment-resistant hypertension.

Ambulatory blood pressure reductions were shown to be greatest in those with the highest starting ambulatory blood pressures, whilst those with blood pressures in the lowest quartile of baseline ambulatory blood pressure showed little response. Use of aldosterone antagonist did not affect the subsequent blood pressure response after denervation and drug changes after denervation did not appear to account for the blood pressure fall seen in the cohort at follow-up.

## Electronic supplementary material

Below is the link to the electronic supplementary material.

Online Appendix 1 (DOCX 98 kb)
