# Supplementary material for: Renal artery sympathetic denervation: observations from the UK experience
Source: Clin Res Cardiol. 2016 Jan 22;105:544–52. doi: 10.1007/s00392-015-0959-4 (PMC4882343; doi:10.1007/s00392-015-0959-4)
Supplement: Supplementary file 1 — Online Appendix 1 (DOCX 98 kb) [file 392_2015_959_MOESM1_ESM.docx]

**Online Supplementary Appendix. – Further Acknowledgements**

The UK Renal Denervation Affiliation consists of 18 centers, each of which has multi-disciplinary teams that have assisted in data acquisition for this project. The authors would therefore like to express thanks to the following people for their key work in this project:

*Royal Brompton and Harefield Foundation Trust*: Dr Hitesh Patel; Dr Ranil DeSilva; Dr Tito Kabir; Dr Gareth Rosser; Dr Alexander Lyon; Mrs Debra Dempster. *Buckinghamshire Healthcare NHS Trust*: Nicola Bowers. *University Hospital Southampton NHS Trust*: Dr Allan Odurny; Dr Derek Waller; Dr Kirsty Armstrong. *St Thomas' Hospital*: Dr Tarun Sabharwal. *Royal Devon and Exeter Hospital:* Dr John Dean; Professor Nick Bellenger; Professor Anthony Watkinson; Dr Denis Kinsella; Professor Angela Shore; Ms Max Hough; Ms Kim Gooding; Ms Bea Knight. *Glasgow Royal Infirmary, Western Infirmary and Institute of Cardiovascular and Medical Sciences:* Dr E Marie Freel; Dr Giles Roditi; Dr Alison Taylor; Dr Jesse Dawson; Dr Adrian Brady; Dr Ram Kasturi. *Bristol Heart Institute:* Dr Amy E Burchell. *Northern General and Royal Hallamshire Hospitals, Sheffield:* Dr Peter R Jackson; Professor Peter A Gaines; Ms Yvonne Jackson*. East Kent Hospitals NHS Foundation Trust:* Dr Robert N Kaikini. *Barts Health NHS Trust and Barts NIHR Cardiovascular BRU*: Professor Charles J Knight; Professor Anthony Mathur; Dr Ajay Jain; Dr Manish Saxena. *Hull and East Yorkshire Hospitals NHS Trust*: Dr Michael Cunnington; Dr Fayaz Khan. *Royal Bournemouth Hospital*: Dr John Paisey; Dr David Beckett, Dr Tristan Richardson. *Conquest Hospital*: Dr Naushin Hossain; Dr Akhtar Zaman; Dr Tharsi Sarvananthan; Dr Omar Shabir. *Queen Elizabeth Hospital, Birmingham.* Dr Sagar Doshi; Professor Jon N Townend; Dr Graham Lipkin. *Heartlands Hospital, Birmingham*: Dr Richard Watkin; Dr Awais Hameed.
